# Commentary: Autism screening and diagnosis in children with congenital heart disease

**DOI:** 10.3389/fped.2026.1936706

**Published:** 2026-08-31

**Authors:** Murtaza Kamal

**Affiliations:** Department Of Pediatric Cardiology, Paramitha Hospital, Hyderabad, India

**Keywords:** autism spectrum disorder, ASD, congenital cardiac anomaly, M-CHAT-R, neurodevelopment, screening

## Introduction

Congenital heart disease (CHD) is the most common structural birth anomaly. As perioperative mortality has fallen, attention has rightly shifted towards the neurodevelopmental cost of surviving early cardiac surgery. Ontiveros Perez and colleagues make a timely contribution to this literature. They prospectively link Modified Checklist for Autism in Toddlers, Revised with Follow-Up (M-CHAT-R/F) screening at 18–30 months to chart-confirmed autism diagnosis, in a cohort of 94 children who required cardiac surgery before initial hospital discharge (1).

It is worth restating, at the outset, why the distinction between these two outcomes matters. Screening with the M-CHAT-R/F is a brief, parent report questionnaire, typically administered in a primary-care or follow-up-clinic setting to a large, largely unselected group of children; a positive result flags an elevated likelihood of autism, not the condition itself. Diagnosis, by contrast, requires a comprehensive, in-person evaluation. This generally involves a developmental paediatrician, psychologist or child psychiatrist, standardised observational instruments such as the Autism Diagnostic Observation Schedule and a detailed developmental history, before autism spectrum disorder can be confirmed clinically. The two therefore differ not only in accuracy but in the time, personnel, and resources each demands, and this gap sits at the centre of several issues raised below.

Their headline finding is striking: a screen positive rate of 14.6%, and a diagnosis rate of 11.7%, both more than three fold the current general-population estimate (2). This reinforces a signal that has been accumulating across the congenital cardiology literature for a decade. The design has real strengths, particularly its reliance on a defined referral population and on chart verified outcomes, rather than on screening results alone. Several features of the data, nonetheless, warrant closer scrutiny before the findings are translated into practice recommendations, especially for cardiology programmes that lack the structured High-Risk Infant Follow-Up (HRIF) infrastructure that made this study possible.

## A small denominator driving large effects

Of the 94 eligible children, only 48 were screened and only 7 screened positive. The confirmed autism subgroup within the screened cohort comprised just six children. Sensitivity of 66%–67% therefore rests on the reclassification of a single case, and its reported confidence interval, 0.33–1.00, spans almost the entire plausible range (1). The authors are appropriately candid about this. Still, a bare comparison against general population prevalence (2) risks conveying more precision than the underlying numbers can bear.

Table 1 places the study's psychometrics beside published benchmarks. What is notable is not that this CHD sample under-performed the general population norming cohort (3); it did not. Rather, its specificity and negative predictive value were unusually high relative to most high risk validation samples. In those samples, the customary trade-off usually runs the opposite way: sensitivity rises while specificity falls, because co-occurring developmental delay inflates false positives (4, 5). A plausible explanation here is verification bias. Referral for full evaluation was left to clinician and family discretion, and 46 of 94 eligible children were never screened at all. Without a fixed, non-discretionary protocol, both the numerator and the denominator of these estimates are shaped by judgements the study cannot audit.

**Table 1:** Reported psychometric performance of the M-CHAT-R/F across study populations`.

Study population	Sensitivity	Specificity	PPV	NPV
CHD cohort, screened subgroup — Ontiveros Perez et al. (1)	66%–67%	92%–93%	57%	95%
General low-risk norming sample — Robins et al. (3)	83%–85%	95%–99%	46.5%	Not reported
Pooled meta-analysis, high-risk subgroup — Aishworiya et al. (4)	82.6%	45.7%	Higher than low-risk subgroup	72.5%
Large real-world primary-care cohort — Harper et al. (5)	38.8%	94.9%	14.6%	98.6%

Values drawn from the cited published abstracts; ranges reflect pre- and post-follow-up interview scoring where reported.

## The phenotypic-overlap problem

A second concern is conceptual, rather than statistical. Children with CHD, independent of any autism diagnosis, commonly display weaknesses in theory of mind, pragmatic language and social cognition. These traits can phenotypically resemble autism without meeting full diagnostic criteria (6). The M-CHAT-R/F was normed on low risk and preterm populations, not on children who have undergone cardiopulmonary bypass, deep hypothermic circulatory arrest or repeated infant general anaesthesia. These exposures plausibly affect the very social communication domains the instrument probes, independent of core autism biology (6, 7).

Read against this background, the study's association between Risk Stratification for Congenital Heart Surgery-2 (RACHS-2) score and autism diagnosis is biologically plausible, but not unambiguous. Greater surgical complexity predicts both closer follow-up and therefore more opportunities for detection and greater hypoxic ischaemic exposure, a genuine risk factor in its own right. A single centre study of this size cannot separate ascertainment from causation, and few current alternatives can either.

## Insurance status as a socioeconomic proxy

The authors attribute the association between public insurance and autism diagnosis to California's disability linked insurance eligibility pathways, rather than to true socioeconomic disadvantage (1). This is a reasonable, locally grounded reading. It is also a reminder that “public insurance” is not a portable proxy for socioeconomic status across health systems. In settings structured differently, including most cardiac referral centres outside the United States, this covariate would carry a different meaning entirely. The conclusion, therefore, should not be imported along with the variable.

## Implications beyond a single referral network

The HRIF model is valuable, but it is not universally available infrastructure. Cognitive and developmental impairment in CHD spans multiple domains and tends to worsen with age (8). Yet, in many referral centres, particularly in resource-constrained settings, follow-up after infant cardiac surgery remains predominantly cardiological, with limited routine access to structured developmental surveillance. If autism prevalence in children with severe CHD genuinely runs three fold higher than in the general population, as this and prior work suggest (1, 4), the practical bottleneck is not simply the decision to screen. It is the referral capacity that a positive result demands, given a positive predictive value of only 57% in this cohort (1). A screening programme without matching evaluation capacity risks generating parental anxiety, without improving outcomes.

## Conclusion

Ontiveros Perez et al. add welcome toddler period data to a still thin literature. Their central message, that CHD severity requiring early surgery tracks with meaningfully elevated autism likelihood, is consistent with the field (1, 4, 6). The specific psychometric estimates, however, rest on a small, clinician selected subgroup, and are better treated as hypothesis generating than confirmatory. Larger, multisite cohorts, using universal, non-discretionary screening and spanning varied insurance and resource settings, are needed before the M-CHAT-R/F's performance in structural heart disease can be considered established. Until then, the more defensible clinical takeaway is not a specific cutoff, but the underlying principle: children surviving early, complex cardiac surgery warrant systematic developmental surveillance, delivered through pathways equipped to act on what screening finds.
